# Serum metabolomics analysis of patients with chikungunya and dengue mono/co-infections reveals distinct metabolite signatures in the three disease conditions

**DOI:** 10.1038/srep36833

**Published:** 2016-11-15

**Authors:** Jatin Shrinet, Jayanthi S. Shastri, Rajni Gaind, Neel Sarovar Bhavesh, Sujatha Sunil

**Affiliations:** 1 Vector Borne Disease group, International Centre for Genetic Engineering and Biotechnology (ICGEB), Aruna Asaf Ali Marg, New Delhi, 110067, India; 2 Transcription Regulation group, International Centre for Genetic Engineering and Biotechnology (ICGEB), Aruna Asaf Ali Marg, New Delhi, 110067, India; 3 T. N. Medical College & B. Y. L. Nair Charitable Hospital, Dr. A. L. Nair Road, Mumbai, 400008, India; 4 Vardhman Mahavir Medical College-Safdarjung Hospital, New Delhi, 110029, India

## Abstract

Chikungunya and dengue are arboviral infections with overlapping clinical symptoms. A subset of chikungunya infection occurs also as co-infections with dengue, resulting in complications during diagnosis and patient management. The present study was undertaken to identify the global metabolome of patient sera infected with chikungunya as mono infections and with dengue as co-infections. Using nuclear magnetic resonance (NMR) spectroscopy, the metabolome of sera of three disease conditions, namely, chikungunya and dengue as mono-infections and when co-infected were ascertained and compared with healthy individuals. Further, the cohorts were analyzed on the basis of age, onset of fever and joint involvement. Here we show that many metabolites in the serum are significantly differentially regulated during chikungunya mono-infection as well as during chikungunya co-infection with dengue. We observed that glycine, serine, threonine, galactose and pyrimidine metabolisms are the most perturbed pathways in both mono and co-infection conditions. The affected pathways in our study correlate well with the clinical manifestation like fever, inflammation, energy deprivation and joint pain during the infections. These results may serve as a starting point for validations and identification of distinct biomolecules that could be exploited as biomarker candidates thereby helping in better patient management.

Dengue (DEN) and chikungunya (CHIK) are among the most important vector borne diseases, together affecting about 40 million people globally^1^,^2^,^3^. Dengue is an acute systemic viral disease that has established itself globally in both endemic and epidemic transmission cycles^4^. With more than one-third of the world population living in areas at risk of this infection, dengue virus is a leading cause of illness and death in the tropics and subtropics^5^. Global distribution map of dengue has estimated and predicted India to be the worst affected^6^. In India dengue contributes about 34% of the total global infections making it a transmission hub^7^. Known to occur as either single infections or as co-infections with dengue^8^,^9^, Chikungunya virus (CHIKV) poses a serious threat to public health^10^ with spread to many parts of the globe^11^. In the Indian sub-continent, while as many as 1.2 million individuals were affected by CHIK in 2006^12^, recent trends have shown it be declining though numbers are still large^13^.

One of the main challenges that clinicians face during diagnosis of CHIK and DEN are overlapping symptoms of these two infections resulting in gross misdiagnosis and patient mismanagement^14^. Recent studies have shown that almost ten percent of suspected dengue cases are in fact co-infected with chikungunya virus making it imperative to distinguish the difference between the two infections for better patient management^15^. Additionally, misdiagnosis also obstructs the true incidence of chikungunya within a region thereby providing incorrect information of epidemiology of the disease and evolution of the virus. There are infection associated independent markers available for chikungunya and dengue and they have been studied to follow progression of chikungunya from its acute to chronic phase^16^, and the progression of dengue from uncomplicated dengue to its more severe conditions^17^,^18^. However, few studies have been devoted to understand the underlying differentiating features between these two infections^15^,^19^.

Metabolomics quantitatively analyses differential regulation of metabolites when living system is perturbed with disease condition or a change in life style. In recent times metabolic profiling of biofluids has provided additional information to gain insight into disease prognosis and to develop diagnostic biomarkers^20^,^21^,^22^. Among the various tools used for metabolomics, nuclear magnetic resonance (NMR) spectroscopy has emerged as an useful and powerful method due to its noninvasiveness, rapidity, reproducibility, need for small sample volumes, and high accuracy in metabolic profiling^23^,^24^. NMR based metabolomics approach has been used to understand the metabolic dysregulation in humans upon viral infections like hepatitis^25^,^26^,^27^ and HIV^28^,^29^.

The present study was undertaken to identify the global metabolome of patient sera infected with chikungunya as mono-infections and with dengue as co-infections. Metabolome from sera collected from healthy individuals and dengue patients was ascertained and used to compare with chikungunya mono/co-infected metabolome. The cohorts were stratified on the basis of age and onset of fever. Chikungunya patient sera metabolites and their pathways were analyzed to correlate with chronic phase of infection. Unique metabolite signatures were identified for chikungunya mono-infections and co-infections with dengue.

## Results

A total of fifty-three samples (Healthy: fifteen, chikungunya (CHIK): fifteen, Dengue (DEN): eleven, chikungunya/dengue co-infection (C/D): twelve) were analyzed. The basic clinical features of the samples are shown in Table 1.

All samples were subjected to ELISA to test for CHIKV-IgM and RT-PCR for detecting CHIKV RNA. Out of fifteen samples of CHIK, all samples were positive by RT-PCR and three of these samples were positive by CHIKV-IgM test as well. In case of DEN samples, two samples tested positive by both CHIKV-IgM test and RT-PCR, three samples were positive only for IgM and remaining samples were found to be positive by RT-PCR. Similarly in co-infection samples, one sample tested positive by CHIKV-IgM and DENV-IgM, three samples were positive by RT-PCR (CHIK), DENV-IgM and RT-PCR (DEN), five samples showed positive result by RT-PCR (CHIK) and DEN-IgM and all other samples were found to be positive by RT-PCR method for both the viruses.

All samples were grouped according to their respective conditions and were analyzed using MetaboAnalyst tool^30^. These samples contained a total of 10722 peaks of metabolites with an average of 202.3 peaks per sample. Proximal peaks are grouped together based on their position using a moving window of 0.03 ppm and a step of 0.015 ppm. Peaks of the same group were summed if they are from one sample. Peaks appearing in less than half of samples in each group were ignored. Missing variables were imputed using SVDIMPUTE. Row-wise normalization was performed using sample Median. Data transformation was done by log normalization method and autoscaling algorithm was used for data scaling. Univariate and multivariate analyses were performed and the significant results (p-value < 0.05) were further subjected to pathway analysis.

### Global profiling of metabolites from CHIK, DEN and CHIK/DEN co-infected sera and pathway analysis

**Figure 1:**
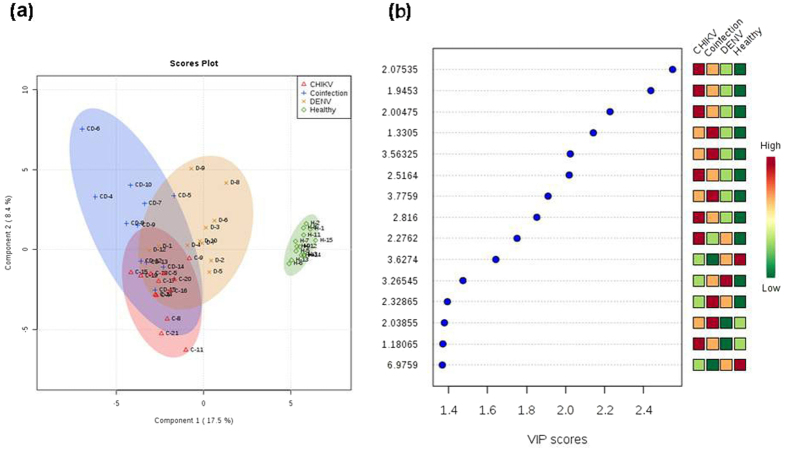
The figure represents the score plot and important metabolites. (**a**) Score-plot of the PLS-DA analysis. (**b**) Important metabolites selected on the basis of VIP score.

Global profiling of metabolites of CHIK, DEN and co-infected serum samples were performed and compared with uninfected samples as control. Totally, fifty three samples containing 10722 peaks with an average of 202 peaks per sample were analyzed. A total of 149 peak groups were formed. One-way analysis of variance (ANOVA) was performed to recognize the significance of the sample cohorts analyzed, followed by post-hoc analyses using Fisher’s LSD to identify the differences among the levels in the datasets. ANOVA analyses identified sixty significant features with p-value < 0.05. PLS-DA and PCA analysis of the datasets were performed to study the variance in the data amongst the five components that were derived upon these analyses. In PLS-DA analysis, Component 1 showed 17.5% variance, Component 2 with 8.4%, Component 3 (8%), Component 4 (5.8%) and Component 5 showed 5.1% variance. The score plot between Component 1 and Component 2 is shown in Fig. 1a. Analysis also identified fifteen important features on the basis of VIP scores (Fig. 1b). 2D scoresplot of the PCA analysis is shown in Supplementary Fig. 1.

Pair-wise analysis between healthy and the three disease conditions were performed to arrive upon the significantly regulated metabolites. In case of CHIK infection, eleven compounds showed considerable regulation; out of which, nine metabolites (Azelaic acid, Mandelic acid, Methylguanidine, D-Maltose, Ethanol, 2-Hydroxycaproic acid, Gluconolactone, Carnitine, Galactitol) were up-regulated with log2(FC) > 2 and p-value < 0.05; levels of two metabolites (Ethanolamine, 1,3-Diaminopropane) were found to be substantially low (Supplementary Table 1). Significant pathways (p-value < 0.05) are shown in Table 2. In case of pathways regulated in CHIK, Glycine, serine and threonine metabolism, Galactose metabolism and Citrate cycle (TCA cycle) pathway showed significant p-value of 1.23E-04, 8.27E-04 and 0.018921 respectively.

Similarly in DEN, fourteen compounds showed substantial regulation, out of these, twelve metabolites were showing increase in their levels (log2(FC) > 2; p-value < 0.05) and two, namely, N-Methyl-a-aminoisobutyric acid and Sarcosine were found to be present in reduced levels when compared to healthy set. Pathway analysis revealed six pathways namely, Glycine, serine and threonine metabolism (p-value = 8.16E-05), Galactose metabolism (p-value = 0.0072443), Starch and sucrose metabolism (p-value = 0.012559), Glyoxylate and dicarboxylate metabolism (p-value = 0.012559), Pentose phosphate pathway (p-value = 0.0394) and Propanoate metabolism (p-value = 0.046418) to be significantly regulated (Table 2).

In case of C/D, twenty compounds showed substantial regulation; out of these, ten metabolites were showing increase in their levels (log2(FC) > 2; p-value < 0.05) and ten were found to have reduced levels of concentration. Pathway analysis revealed that eight pathways namely, Glycine, serine and threonine metabolism (p-value = 2.50E-04), Galactose metabolism (p-value = 0.0014424), Pyrimidine metabolism (p-value = 0.0058899), Fructose and mannose metabolism (p-value = 0.020719), Glyoxylate and dicarboxylate metabolism (p-value = 0.02309), Citrate cycle (TCA cycle) (p-value = 0.024828), Alanine, aspartate and glutamate metabolism (p-value = 0.034974), beta-Alanine metabolism (p-value = 0.046453) were differentially regulated (Table 2).

### Role of age on regulation of metabolites and their pathways during mono and co-infection

Age has an impact on disease progression in infections like chikungunya. Geriatric population has been reported to be affected in a more pronounced manner with a prolonged chronic phase as compared to individuals of younger age affected by the infection^31^. Our sample cohort in the present study also revealed that the median age was the maximum in case of CHIK (Table 1). To understand the impact age may have on the metabolite profiles; we studied our metabolite data sets on the basis of age in the three disease conditions.

**Figure 2:**
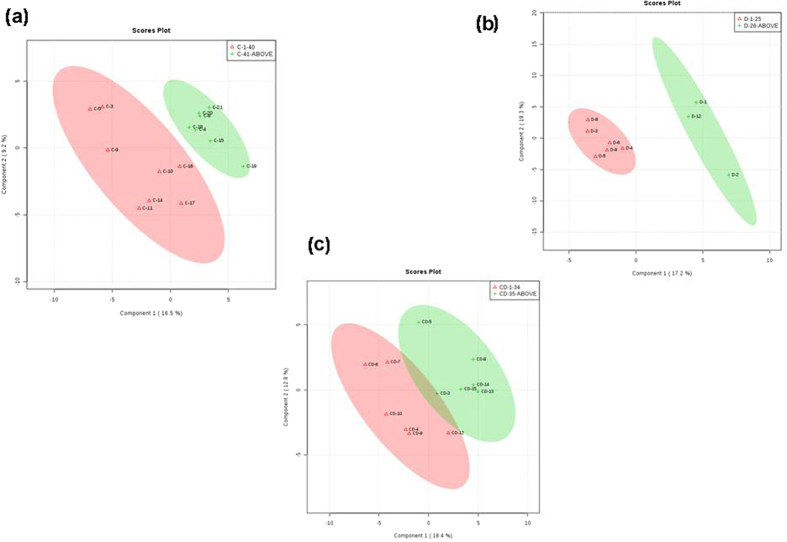
The figure represents the score plots of PLS-DA analysis of different age group. (**a**) PLS-DA score plot of CHIK. (**b**) PLS-DA score plot of DEN. (**c**) PLS-DA score plot of co-infection.

CHIK samples were divided into two age groups, 12–40 years and 41–70 years. Fold change (FC) analysis of the data identified nineteen important features whereas *t*-test analysis predicted five important features with p-value < 0.05. PCA analysis revealed that principal component 1 (PC1) showed maximum variation i.e., 22.2%, followed by PC2 (18.7%), PC3 (14.5%), PC4 (9.9%) and PC5 (6.8%) (Supplementary Fig. 2). In PLS-DA analysis, all the five components also showed the separation pattern similar to PCA analysis, i.e., component 1 showed 16.5% variance, component 2 with 9.2%, component 3 (11.8%), component 4 (13.7%) and component 5 showed 12% variance (Fig. 2a). In CHIK dataset, three compounds (Trimethylamine, Putresine and Mevalonic acid) showed regulation; out of these, two metabolites (Trimethylamine, Putresine) were showing increase in their levels (log2(FC) > 2 and p-value < 0.05) and levels of Mevalonic acid was found to be substantially low (Supplementary Table 2) in age group (41–70). D-Glutamine and D-glutamate metabolism and Alanine, aspartate and glutamate metabolism showed significant p-value of 0.022661 and 0.04891 respectively (Table 3).

DEN samples were divided into two age groups, 12–25 years and 26–51 years. Fold change (FC) analysis of the data identified 35 important features whereas *t*-test analysis predicted seven important features with p-value < 0.05. PCA and PLS-DA analysis of the samples were performed to study the variance in the data. In PCA analysis, principal component 1 (PC1) showed maximum variation i.e., 25.8%, followed by PC2 (18.7%), PC3 (15.9%), PC4 (11.9%) and PC5 (10.6%) (Supplementary Fig. 2). In PLS-DA analysis, component 1 showed 17.2% variance, component 2 with 18.3%, component 3 (14.3%), component 4 (12.5%) and component 5 showed 11.6% variance (Fig. 2b). In the DEN dataset, two compounds namely, Capric acid and Sarcosine showed substantial regulation, Capric acid showed increase in its levels with log2(FC) > 2 and p-value < 0.05 and levels of Sarcosine was found to be substantially low (Supplementary Table 2) in age group (26–51). The pathways with p-value < 0.05 are shown in Table 3. Tyrosine metabolism and Alanine, aspartate and glutamate metabolism showed significant p-value of 0.0092504 and 0.04891 respectively.

Co-infected samples were divided into two age groups, 12–34 years and 35–60 years. Fold change (FC) analysis of the data identified 23 important features whereas t-test analysis predicted three important features with p-value < 0.05. The Volcano plot analysis results are shown in Supplementary Table 2. In PCA analysis, principal component 1 (PC1) showed maximum variation i.e., 25%, followed by PC2 (12.8%), PC3 (15.5%), PC4 (9.1%) and PC5 (8.3%) (Supplementary Fig. 2). In PLS-DA analysis, component 1 showed 18.4% variance, component 2 with 12.8%, component 3 (15.5%), component 4 (9.1%) and component 5 showed 6% variance (Fig. 2c). In the co-infection group, six compounds namely, 3-Hydroxyphenylacetic acid, Pyridine, Gentisic acid, Epinephrine, Atrolactic acid and 4-Hydroxyphenylpyruvic acid showed substantial regulation and all of them were showing increase in their levels (log2(FC) > 2 and p-value < 0.05). Pathway analysis (Table 3) revealed that Tyrosine metabolism is significantly affected (p-value = 2.99E-05).

### Role of fever duration on regulation of metabolites and their pathways during mono and co-infection

One important clinical symptom in CHIK and DEN is fever. We sort to understand alterations in the metabolites in a scenario of fever duration at the time of patient reporting for sample collection in both mono-infections and co-infections. For this purpose, all the categories (CHIK, DEN and C/D samples) were combined and then divided into two groups on the basis of fever duration at the time of sample collection from the patients. The first group comprised of samples that were collected when the fever presentation was within the first five days as per the clinical history sheet and those samples that were collected when the patient came to the clinic with fever for more than five days (6–12 days) comprised the second group. ANOVA analyses were performed for each group separately. Twenty seven significant features with p-value < 0.05 were identified in case of first group (1–5 days) and in case of second group, only fourteen features were found to be significant (p-value < 0.05) (Table 4).

**Figure 3:**
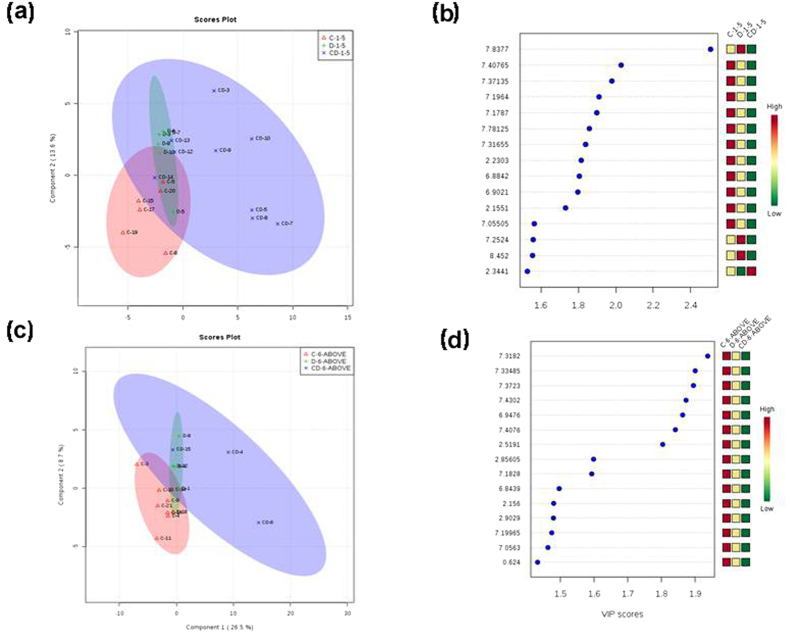
The figure represents the score plot and important metabolites. (**a**) PLS-DA score plot of fever duration 1–5. (**b**) Important metabolites selected on the basis of VIP score. (**c**) PLS-DA score plot of fever duration 6–12. (**d**) Important metabolites selected on the basis of VIP score.

PLS-DA analysis of both the group revealed similar pattern of variance (Fig. 3a,c). The fifteen important features each identified by PLS-DA analyses on the basis of VIP scores for both the group were further analyzed for the identification of their respective pathways (Fig. 3b,d). The pathway analysis of group with fever duration 1–5 days revealed four significant pathways namely, Phenylalanine, tyrosine and tryptophan biosynthesis (p-value = 0.00746), Ubiquinone and other terpenoid-quinone biosynthesis (p-value = 0.01307), Nitrogen metabolism (p-value = 0.01524) and Phenylalanine metabolism (p-value = 0.02003) (Table 4). Pathway analysis of the group with fever duration 6–12 days revealed that Tyrosine metabolism pathway was regulated with less significance (p-value = 0.0613).

### Pathways affected by intensity of joint involvement in CHIK

One of the delineating features between chikungunya and dengue is the involvement of bone-joints. Chikungunya patients are affected by acute arthralgia. In this context, we studied chikungunya mono-infections and co-infections with dengue in the context of bone/joint involvement. Dengue samples could not be analyzed as all samples presented with non-restricted movement and could not be categorized pair-wise for the analysis.

**Figure 4:**
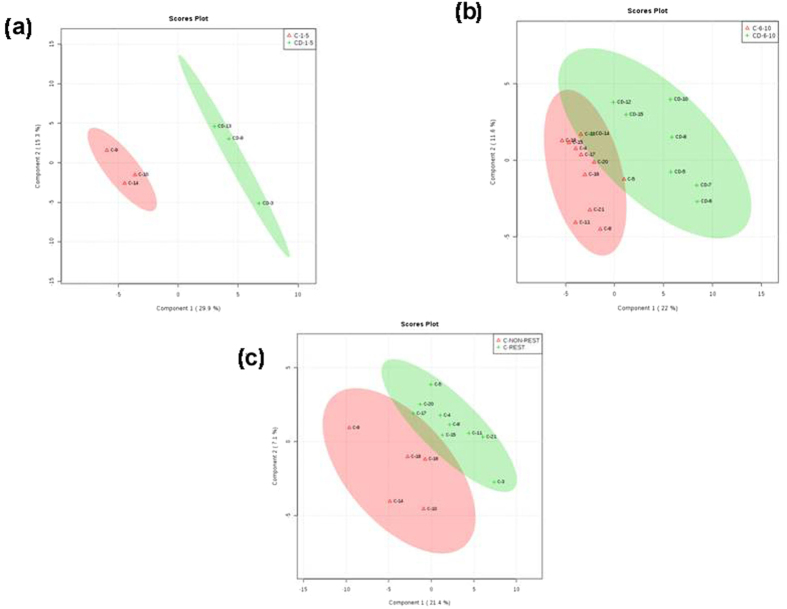
The figure represents the score plots of PLS-DA analysis. (**a**) PLS-DA score plot of VAS Score 1–5. (**b**) PLS-DA score plot of VAS score 6–10 (**c**) PLS-DA score plot of movement (restricted and non-restricted) in CHIK infection.

CHIK samples were divided into two groups according to restricted and non-restricted movement. Fold change (FC) analysis of the data identified thirteen important features whereas *t*-test analysis predicted 3 important features with p-value < 0.05. In PLS-DA analysis, all the five components also showed the separation pattern, i.e., component 1 showed 21.4% variance, component 2 with 7.1%, component 3 (9.9%), component 4 (12.2%) and component 5 showed 11.6% variance (Fig. 4a). Total of two pathways namely, Pantothenate and CoA biosynthesis and β-Alanine metabolism were differentially regulated significantly with p-value of 0.033289 and 0.034508 respectively.

### Role of VAS score on metabolites and their pathways during CHIK infection and co-infection

Chikungunya exists in two stages, the febrile acute phase and the chronic arthritic phase. The arthritic phase is evaluated by the prolonged and chronic swelling in the bones that is assessed by the visual analog score (VAS). A higher VAS reflected higher chronicity and a more severe case presentation of the disease. We analyzed CHIK mono-infections and the co-infected samples for metabolite differences on the basis of their VAS. CHIK and co-infection samples were divided into two groups, first group with the VAS score 1–5 and second group with VAS score of 6–10. PLS-DA analyses of both the sets were performed separately (Fig. 4b,c). As only two datasets (CHIK and C/D) were used for analyses, Volcano test was performed to identify significant metabolites. Seven significant features (p-value < 0.05) were identified in case of first group (VAS = 1–5) and in case of second group (VAS = 6–10), only fourteen features were found to be significant (p-value < 0.05). These significant metabolites were further analyzed for identification of pathways. The pathway analysis of group with VAS score 1–5 showed two significantly regulated pathways. Pentose phosphate pathway and Galactose metabolism were found to be significant, each with p-value 0.008741 and 0.01413 respectively. Similarly, in case of VAS 6–10, five pathways were found to be significant namely, Phenylalanine metabolism (p-value = 9.32E-05), Tyrosine metabolism (p-value = 0.00857), Phenylalanine, tyrosine and tryptophan biosynthesis (p-value = 0.01015), Ubiquinone and other terpenoid-quinone biosynthesis (p-value = 0.017682) and Nitrogen metabolism with p-value of 0.020591 (Table 5).

### Regulation of pathways and important metabolites during CHIK mono and co-infection with DEN

**Figure 5:**
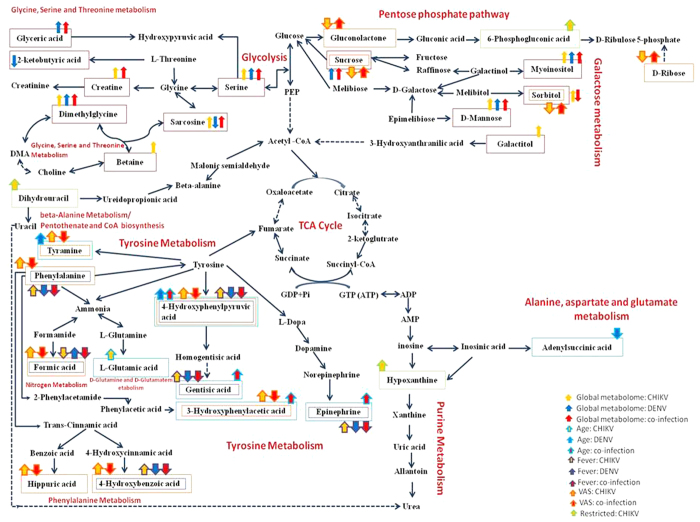
The figure represents the important metabolites identified in this study. The red box represents the metabolite detected in global metabolomic study, blue box represents metabolites detected from the study of role of age during infection, orange and purple boxes represents metabolites from VAS and role of fever study and the metabolites detected while studying the role of joint movements are represented by green box. The descriptions of arrows are provided as legend in the figure.

Analyzing the pathways that were significantly perturbed in our study prompted us to develop a model to provide a link between the various pathways and is depicted as Fig. 5. It was seen that Glycine, serine and threonine metabolism and Galactose metabolism are the most perturbed pathways in all three disease conditions (p-value < 0.01). In those analyses involving specific conditions like fever, age and involvement of joint movements, tyrosine metabolism and phenylalanine metabolism were found to be the most affected.

**Figure 6:**
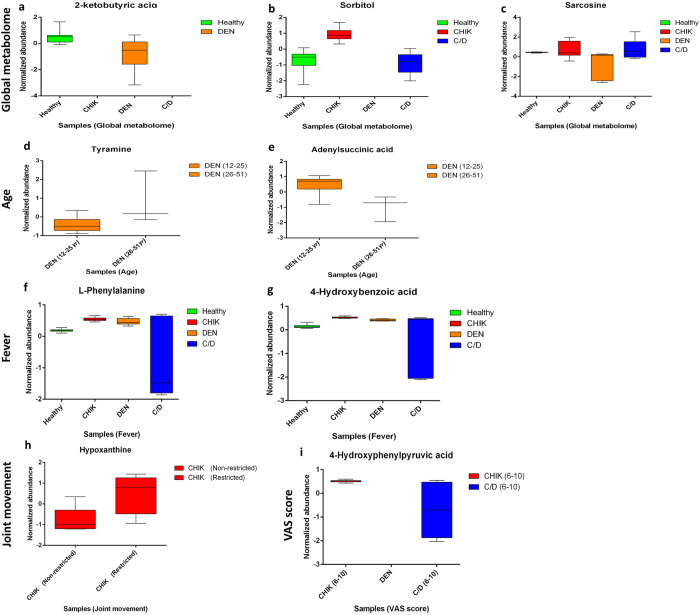
The figure represents Box and Whisker plots of representative metabolites detected during different analysis. Y-axis shows normalized concentrations of metabolites.

Additionally, we have observed that several metabolites were differentially regulated in the diseased conditions in the present study. To understand the significance of this regulation amongst the metabolites, we selected those metabolites that were highly regulated from our analyses and plotted them on the basis of their presence in the various conditions to hypothesize if they could used as biomolecules specific to that condition (Fig. 6a–i). The results are being discussed in detail in the following section.

## Discussion

Chikungunya and Dengue viruses belong to different families but share common mammalian host and vectors. Several studies have reported the co-occurrence and co-infection of these viruses in humans^14^,^32^. Diagnosis of these two infections is difficult owing to overlapping clinical presentation leading to misdiagnosis. The situation is further complicated during co-infection of these two infections leading to poor clinical management of the patients. Previous studies have been divided with respect to the clinical outcome in cases of mono and co-infections. While some studies have reported no differences in symptoms or clinical outcome between the two conditions^9^,^33^, others have shown distinct differences in clinical symptoms between mono and co-infections^8^,^15^. One of our recent study has revealed distinct clinical differences between chikungunya mono-infections and co-infections with dengue and shows that severe arthralgia and involvement of the small joints are more pronounced in co-infections as compared to CHIK mono-infections and hardly present in dengue mono-infections^15^. At a molecular level, differences in symptoms in CHIK mono-infections and in co-infections with dengue indicate regulation of metabolic pathways and their products. In case of dengue, several studies have been conducted utilizing serum metabolomics to identify differentiating small molecules in different type of dengue^34^,^35^. The present study was conducted to evaluate differential regulation of metabolite in patients affected by CHIK as mono-infections and with DEN as co-infections. Our study confirms that serum metabolome is distinctly perturbed in chikungunya mono-infection as well as in chikungunya co-infection with dengue.

With respect to the perturbed pathways, we identified that Glycine, serine and threonine metabolism and Galactose metabolism are the most perturbed pathways in all three disease conditions (Fig. 6). A study analyzing effect of DEN infection in endothelial cell lines corroborate our findings^36^ while another study emphasizes on the importance of glycolysis for efficient virus replication in the host^37^. Disruption of important metabolites like D-serine, sorbitol, mannose directly impact other connected pathways like the PPP required for metabolism. When we study the pathways in specific conditions like fever, age and involvement of joint movement, we show that tyrosine metabolism and phenylalanine metabolism are most affected. Dysregulation of Tyrosine metabolism has been reported with age and arthritic conditions^38^,^39^. Involvement of these pathways in different infections like HIV has been previously reported^40^,^41^. Interestingly Pyrimidine metabolism is also affected during the co-infection which could possibly affect the nucleic acid synthesis and affect cell cycle. Overall TCA cycle is also affected in both mono and co-infections which could lead to energy deprivation among the patients.

One of the purposes of such studies is identification of molecules that could be exploited for biomarker development. In this study we attempted to identify metabolites that were significantly regulated between the three disease conditions. For instance, 2-ketobuyric acid was totally absent in the case of CHIK and C/D and was found to be down-regulated in case of DEN as compared to the healthy set (Fig. 6a). Known to be involved in the degradation of threonine and also be converted to succinyl CoA and enter the citric acid cycle, this compound has been shown to be involved in the plant defense^42^. However, sorbitol was found to be totally absent in DEN group and found to be up-regulated in case of CHIK mono-infections and down-regulated in case of C/D (Fig. 6b). Sorbitol has been reported to participate in apoptosis through caspase activation and cytochrome c release^43^ and its role in such a distinct manner in the present infections warrants in-depth studies. Similarly, in case of sarcosine, the levels were significantly varied when compared to healthy set, with DEN showing significant down-regulation as compared to the other two disease conditions (Fig. 6c). Sarcosine has been reported in other disease conditions ranging from HIV to cancer^40^,^44^. With respect to age, tyramine and adenylsuccinic acid were significantly regulated (Fig. 6d,e). Tyramine is known to be associated with increasing age and acts by releasing endogenous norepinephrine which elevates metabolism and is indirectly proportional in its synthesis with age^45^,^46^. In case of fever, compounds like L-phenylalanine and 4-hydroxybenzoic acid was found to highly down-regulated in case of C/D patients (Fig. 6f,g) clearly emphasizing the effect co-infection with the two viruses has on amino acid metabolism. L-phenylalanine has been previously reported to be regulated in case of classic dengue patients^47^. More specifically, phenylalanine/tyrosine ratio is considered to be of clinical relevance in some disease conditions^41^,^48^,^49^.

With respect to bone-joint involvement in the three disease conditions, it is known that in dengue there is minimal joint involvement while involvement of the small joints is seen in chikungunya mono-infections and when co-infected with dengue^15^. Along with restriction of the joints that is an indication as to the severity of chikungunya, visual analogue scale (VAS) score is another measure to examine the impairment on the joints and the VAS score gives an indication as to the chronicity of chikungunya. As viral induced arthritis is an important feature of chikungunya, it is important to understand the disruption in the metabolic pathways involved in this condition. Purine metabolism is known to be disrupted in inflammatory arthritis^50^,^51^ and among the significant metabolites in this pathway, hypoxanthine, xanthine and uric acids are important. Known to be low in normal physiological conditions, hypoxanthine levels increase upon tissue damage. In the present study, hypoxanthine was found to be up-regulated in our study in chikungunya patients who displayed highly restriction in their joints (Fig. 6h). Similarly, studying the metabolite profile of patients with high VAS score revealed regulation of 4-Hydroxyphenylpyruvic acid which is an intermediate in tyrosine metabolism (Fig. 6i) and has been implicated in rheumatoid arthritis owing to its interaction with the macrophage migration inhibitor factor (MIF), a secreted protein that activates macrophages^52^.

## Conclusion

Our study has thrown light to global metabolome of patient sera infected with chikungunya as mono-infections and with dengue as co-infections. Analysis of the sera metabolome revealed that distinct metabolites were deregulated in distinct disease conditions. Further stratification of the cohorts on the basis age, onset of fever and joint involvement revealed that specific pathways were involved in these metabolic functions and disease-specific metabolites were identified. Further validations of these metabolites over a larger patient population may pave way to identification of distinct biomolecules that could be exploited for differential diagnosis thereby helping in better patient management.

## Materials and Methods

### Ethics Clearance

Sera samples were obtained from a cohort of samples that were recruited as part of two multicentric longitudinal studies, one study on divergence of chikungunya in India and the other, evaluation of chikungunya and dengue co-infections. Individuals older than 12 years were recruited in the study upon their written informed consent based on the information in the patient information sheet. In case of participants lesser than 18 years, consent was obtained from their parents/guardians. The studies were approved by the Institutional ethical review board at all the three participating institutes, namely, Vardhman Mahavir Medical College-Safdarjung Hospital, New Delhi (VMMC/SJH/2013/13, IEC/VMMC/SJH/2014/575), BYL Nair hospital, Mumbai (No:153) and ICGEB (ICGEB/IEC/2010/01, ICGEB/IEC/2014/01). All methods followed in the study were performed in accordance with the approved guidelines.

### Clinical samples

The patients were recruited as part of an ongoing study pertaining to chikungunya and dengue divergence and co-infection status in India. Two hospitals, namely, VMMC and Safdarjung Hospital, New Delhi and BYL Nair hospital, Mumbai were part of this study. Both these hospitals are tertiary care center and important referral centers for infectious diseases. Individuals above 12 years of age who presenting with dengue-like symptoms, including fever or had a history of fever within the last seven days, headache, nausea, vomiting, joint pain, swollen joints, aches and pains, rashes, eye pain or hemorrhagic manifestations were enrolled in this study. Upon enrollment, case record form (CRF) of each patient was filled along with clinical disease activity index (CDAI) and his or her recent travel activity. Whole blood was collected in non-additive and EDTA containing blood collection tubes and immediately processed for sera/plasma. Sera and plasma samples were stored at −80 °C until further use.

The samples were evaluated both serologically as well as through RT-PCR for the presence of CHIK and DEN. Sera samples were tested for CHIK/DEN IgM and DEN NS1 using kits provided by NIV, Pune. Further, nucleic acid was extracted from sera and processed for checking RNA copies in all samples enrolled. Samples testing positive by either/both by the above procedures were grouped into CHIK mono-infection, DEN mono-infection and CHIK/DEN co-infection.

### NMR spectroscopy

All the positive serum samples were processed for ^1^H NMR spectroscopy. 160 μl of NMR buffer were added to 40 μl of serum sample to make the total volume to 200 μl. The NMR experiments were performed at 298 K using BrukerAvance III spectrometer equipped with cryogenic triple-resonance TCI probe, operating at the proton field strength of 500 MHz. The Carr–Purcell–Meiboom–Gill (CPMG) echo sequence was used to partially suppress the protein signals^53^. A total CPMG delay of 300 ms was used with an echo time of 200 ms. All one-dimensional ^1^H NMR spectra were measured with 32 scans with a relaxation delay of 4 sec. For each sample free induction decays (FIDs) were collected with a spectral width of 10,000 Hz and acquisition time of 3.28 sec (t_1max_). The peaks were processed using Topspin 2.1 (Bruker AG, Falländen, Switzerland). The base line correction; peak alignment and scaling of the peaks were performed and referenced to DSS at 0 ppm. The peaks from 6.0–4.5 ppm region were excluded to remove water signals.

### Statistical Data analysis

The peaks were picked for each sample separately and were further analyzed and because of the high dimensionality and complexity of the NMR data, multivariate statistical analysis of the data is required. All the statistical analysis and pathway annotations were carried out using MetaboAnalyst web tool (www.metaboanalyst.ca). MetaboAnalyst accept different type of data format generated either from NMR or MS experiments. The tool performed data integrity check. Missing values were imputated using Singular Value Decomposition (SVDIMPUTE) algorithm. Row-wise data was normalized using sample median, log transformation was performed for normalization of the data and for data scaling autoscaling algorithm was used. For multi group analysis, one way ANOVA was performed followed by post-hoc analyses using Tukey’s HSD. For identifying important features in pairwise data analysis, three test namely; fold-change analysis with threshold value 2, t-test and volcano plot which is combination of former two tests were performed. The features with threshold value >2 and p-value < 0.05 were considered significant. For predicting variance in samples, chemometric analysis like Principal Component Analysis (PCA), Partial Least Squares Discriminant Analysis (PLS-DA) were performed. The NMR peaks found to be significantly modulated were annotated using COLMAR^54^ and MetaboMiner web servers^55^. These servers perform the search for the peaks in Metabolome Data Bank (www.hmdb.ca)^56^ and annotate the peaks by identifying the corresponding metabolites. The significant pathways involved in the infections were also identified using MetaboAnalyst tool.

## Additional Information

**How to cite this article**: Shrinet, J. *et al*. Serum metabolomics analysis of patients with chikungunya and dengue mono/co-infections reveals distinct metabolite signatures in the three disease conditions. *Sci. Rep.* **6**, 36833; doi: 10.1038/srep36833 (2016).

**Publisher’s note:** Springer Nature remains neutral with regard to jurisdictional claims in published maps and institutional affiliations.

## Supplementary Material

Supplementary Information

## Figures and Tables

**Table 1:** Details of clinical samples showing mean age, fever duration and visual analogue scale (VAS) score.

Group	Number of samples	Mean age (year range)	Fever duration (days range)	VAS score
Healthy	15	32 (25–48)	—	—
Chikungunya	15	40 (20–70)	5 (1–12)	7 (4–9)
Dengue	11	25 (10–51)	7 (2–30	NA
Co-infection	12	33 (17–46)	4 (2–7)	5 (3–7)

**Table 2:** Table shows identified significant pathways upon CHIK, DEN and co-infection with p-value < 0.05.

Sample	Pathway Name	Total	Hits	p-value	Compounds
**CHIK**	Glycine, serine and threonine metabolism	48	5	1.23E-04	Betaine, Sarcosine, dimethylglycine, D-serine, Creatine
	Galactose metabolism	41	4	8.27E-04	D-mannose, Sorbitol, Galactidol, Myoinositol
	Citrate cycle (TCA cycle)	20	2	0.018921	Citric acid, cis-actonic acid
**DEN**	Glycine, serine and threonine metabolism	48	5	8.16E-05	Dimethylglycine, Sarcosine, 2-ketobutyric acid, D-Serine, Glyceric acid
	Galactose metabolism	41	3	0.007244	D-Mannose, Myoinositol, Sucrose
	Starch and sucrose metabolism	50	3	0.012559	D-maltose, Sucrose, β-D-Fructose
	Glyoxylate and dicarboxylate metabolism	50	3	0.012559	Glyceric acid, Oxaloacetic Acid, Formic Acid
	Pentose phosphate pathway	32	2	0.0394	Gluconolactone, Glyceric acid
	Propanoate metabolism	35	2	0.046418	2-ketobtyric acid, Methylmalonic acid
**Co-infection (C/D)**	Glycine, serine and threonine metabolism	48	5	2.50E-04	Glyceric acid, D-serine, Creatine, Sarcosine, Dimethylglycine
	Galactose metabolism	41	4	0.001442	D-mannose, Myoinositol, Sucrose, Sorbitol
	Pyrimidine metabolism	60	4	0.00589	L-Glutamine, Dihydrothymine, β-alanine, Malonic acid
	Fructose and mannose metabolism	48	3	0.020719	Sorbitol, Mannitol, D-Mannose
	Glyoxylate and dicarboxylate metabolism	50	3	0.02309	Glyceric acid, Citric acid, Oxaloacetic acid
	Citrate cycle (TCA cycle)	20	2	0.024828	Oxaloacetic acid, Citric acid
	Alanine, aspartate and glutamate metabolism	24	2	0.034974	L-glutamine, Oxaloacetic acid
	β-Alanine metabolism	28	2	0.046453	Malonic acid, β-alanine

**Table 3:** The table represents the significant pathways during infection.

Sample (age)	Pathway Name	Total	Hits	p-value	Compounds
**CHIK (41–70/12–40)**	D-Glutamine and D-glutamate metabolism	11	1	0.022661	L-Glutamic acid
	Alanine, aspartate and glutamate metabolism	24	1	0.04891	L-Glutamic acid
**DEN (26–51/12–25)**	Tyrosine metabolism	76	2	0.0092504	4-hydroxyphenylpyruvic acid, Tyramine
	Alanine, aspartate and glutamate metabolism	24	1	0.04891	Adenylsuccinic acid
**C/D (35–60/12–34)**	Tyrosine metabolism	76	4	2.99E-05	Gentisic acid, 3-Hydroxyphenylacetic acid, Epinephrine, 4-Hydroxyphenylpyruvic acid

The age groups compared for each datasets are shown within the brackets.

**Table 4:** The table represents the significant pathways identified during the study of role of fever duration on infection.

Samples (Days)	Pathway Name	Total	Hits	p-value	Metabolites
**Fever (1–5)**	Phenylalanine, tyrosine and tryptophan biosynthesis	27	2	0.007464	4-Hydroxyphenylpyruvic acid, L-Phenylalanine
	Ubiquinone and other terpenoid-quinone biosynthesis	36	2	0.013067	4-Hydroxybenzoic acid, 4-Hydroxyphenylpyruvic acid
	Nitrogen metabolism	39	2	0.015242	L-Phenyalanine, Formic acid
	Phenylalanine metabolism	45	2	0.020028	L-Phenyalanine, 4-Hydroxybenzoic acid
**Fever (6–12)**	Tyrosine metabolism	76	2	0.061286	Gentisic acid, Epinephrine

**Table 5:** The table represents the significant pathways identified during the study of role of VAS score on infection.

Samples	Pathway Name	Total	Hits	p-value	Metabolite
**VAS (1–5)**	Pentose phosphate pathway	32	2	0.008741	Gluconolactone, D-ribose
	Galactose metabolism	41	2	0.01413	Sorbitol, Sucrose
**VAS (6–10)**	Phenylalanine metabolism	45	4	9.32E-05	L-Phenylalanine, 4-Hydroxybenzoic acid, Hippuric acid, 3-Hydroxyphenylacetic acid
	Tyrosine metabolism	76	3	0.00857	4-Hydroxyphenylpyruvic acid, Tyramine, 3-Hydroxyphenylacetic acid
	Phenylalanine, tyrosine and tryptophan biosynthesis	27	2	0.01015	4-Hydroxyphenylpyruvic acid, L-Phenyalanine
	Ubiquinone and other terpenoid-quinone biosynthesis	36	2	0.017682	4-Hydroxybenzoic acid, 4-Hydroxyphenylpyruvic acid
	Nitrogen metabolism	39	2	0.020591	L-Phenylalanine, Formic acid
