# Supplementary material for: Serum metabolomics analysis of patients with chikungunya and dengue mono/co-infections reveals distinct metabolite signatures in the three disease conditions
Source: Sci Rep. 2016 Nov 15;6:36833. doi: 10.1038/srep36833 (PMC5109290; doi:10.1038/srep36833)
Supplement: Supplementary Information [file srep36833-s1.pdf]

Serum metabolomics analysis of patients with chikungunya and dengue mono/co-infections reveals distinct metabolite signatures in the three disease conditions

Jatin Shrinet[1], Jayanthi S. Shastri[3], Rajni Gaind[4], Neel Sarovar Bhavesh[2]* and Sujatha Sunil[1]*

[1]Vector Borne Disease group, [2]Transcription Regulation group, International Centre for Genetic Engineering and Biotechnology (ICGEB), Aruna Asaf Ali Marg, New Delhi, India 110067

[3]T. N. Medical College & B. Y. L. Nair Charitable Hospital, Dr. A. L. Nair Road, Mumbai, India 400008

[4]Vardhman Mahavir Medical College & Safdarjung Hospital, New Delhi, India 110029

*To whom correspondence should be addressed. E-mail: neelsb@icgeb.res.in, sujatha@icgeb.res.in

Supplementary Figure 1. The figure represents PCA analysis ofCHIKV, DENV and Co-infected sera samples.**(A)**Pairwise score polts of selected PCs. The variance of each PC is shown in corresponding diagonal cells. **(B)**The 2D score plot between selected PCs.

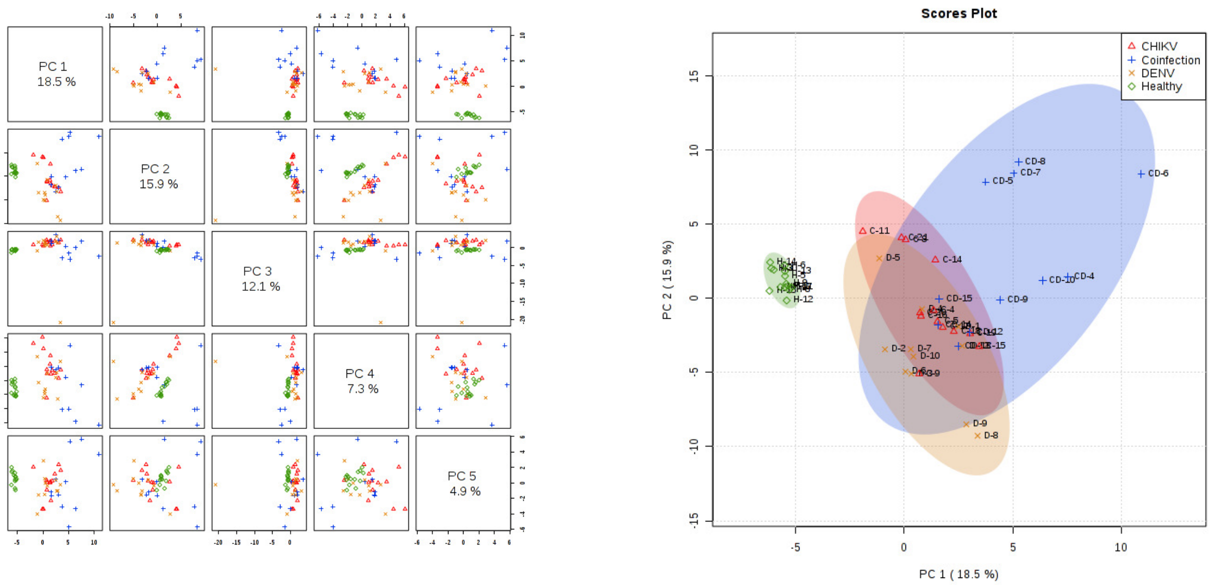

Supplementary Figure 2. The figure represents PCA analysis to study the role of age on the metabolites and the pathways during CHIK, DENV and Co-infection infection.

(A) Pairwise score polts of selected PCs of CHIKV samples. The variance of each PC is shown in corresponding diagonal cells.

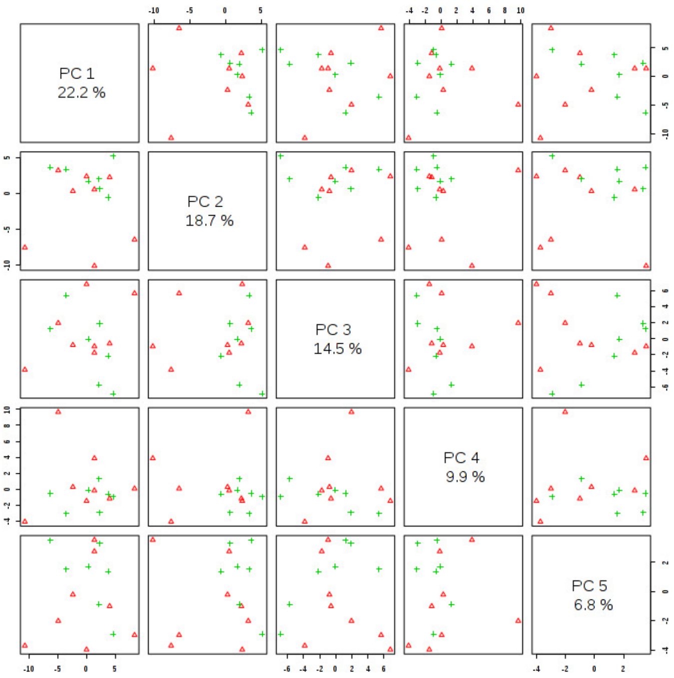

(B) Pairwise score polts of selected PCs of DENV samples. The variance of each PC is shown in corresponding diagonal cells.

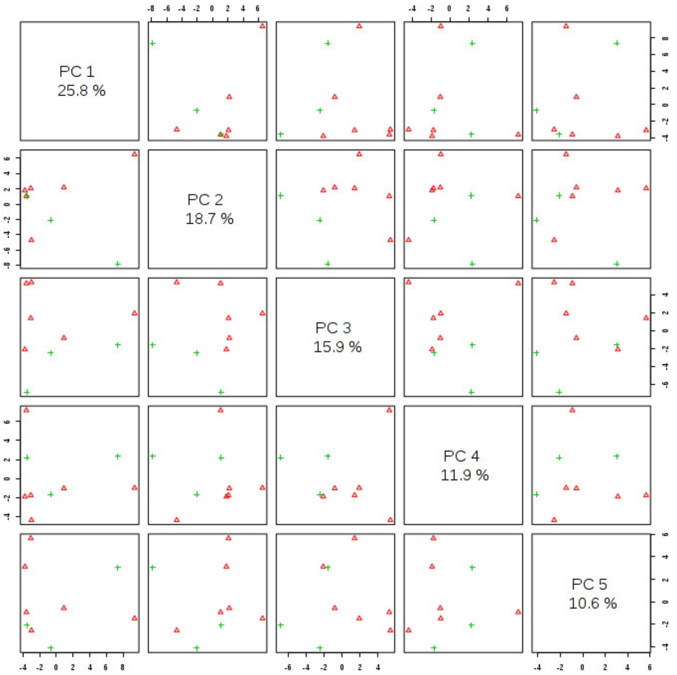

(C) Pairwise score polts of selected PCs of Co-infection samples. The variance of each PC is shown in corresponding diagonal cells.

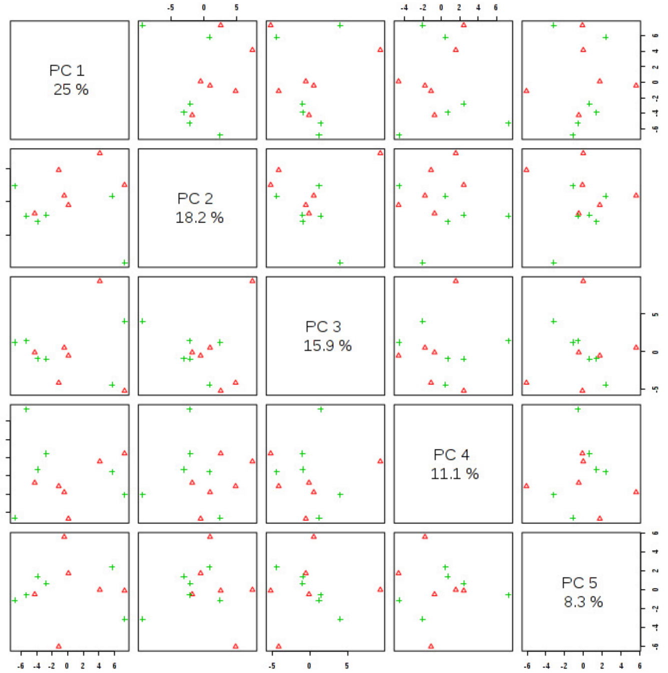

Supplementary Table 1. The table represents the results ofVolcano plot analyses. The significantly regulated compounds with log2(FC) >= ±2 and p-value <= 0.05 upon CHIKV, DENV and Co-infection are represented with asterisk (*) mark.

Samples	Metabolites	ppm	log2(FC)	p-value
**Chikungunya**	Azelaic acid*	4.8208	4.4041	0.042318
	Mandelic acid*	4.99765	3.7286	0.000862
	Methylguanidine*	3.3581	3.6175	0.002021
	D-Maltose*	4.64445	3.039	6.16E-07
	D-Maltose*	5.2388	2.9416	5.99E-11
	Ethanol*	1.1767	2.7009	0.045005
	2-Hydroxycaproic acid*	1.32585	2.6639	1.15E-10
	Gluconolactone*	4.1249	2.0869	0.000728
	Carnitine*	3.4052	2.0594	0.000356
	Galactitol*	3.67275	2.0065	0.017548
	L-Sorbose	3.4925	1.9378	0.022
	Dimethylglycine	3.707	1.9002	0.003645
	cis-Aconitic acid	3.4325	1.8962	0.017873
	D-Mannose	3.56535	1.8007	2.71E-06
	Theophylline	3.5373	1.7966	0.003617
	p-Aminobenzoic acid	7.74175	1.6288	0.025167
	Creatine*	3.9167	1.5357	0.023778
	Sorbitol	3.7651	1.4642	5.38E-08
	D-Serine	3.8275	1.4223	0.005142
	3-Chlorotyrosine	1.4905	1.4059	1.21E-06
	Betaine	3.8897	1.3676	0.046497
	Ethylparaben	7.77755	1.2772	1.96E-06
	Myoinositol	3.2673	1.2545	2.69E-05
	4-Methoxyphenylacetic acid	7.3232	1.0646	1.06E-06
	Sarcosine	3.6013	1.0388	8.21E-05
	2-Hydroxycaproic acid	1.72285	-1.0243	4.25E-06
	5-Hydroxyindoleacetic acid	7.1969	-1.1378	6.75E-07
	Citric acid	2.5452	-1.1495	0.006146
	Citramalic acid	2.4711	-1.208	5.25E-07
	2,2-Dimethylsuccinic acid	2.67885	-1.3474	2.84E-05
	Isopropyl alcohol	4.0001	-1.4102	6.2E-07
	Guanidine	2.26435	-1.6052	1.29E-07
	Sorbitol	3.6318	-1.9676	4.85E-08
	Ethanolamine*	3.1382	-2.2429	1.12E-14
	1,3-Diaminopropane*	3.1119	-3.1049	5.09E-12
**Dengue**	Methylmalonic acid*	3.1848	3.3244	5.37E-05
	Methylglutaric acid*	4.6474	3.1674	2.78E-10
	Sucrose*	3.4625	2.7681	1.57E-09
	D-Maltose*	5.2388	2.7401	2.85E-12
	D-Mannose*	3.3898	2.6141	2.8E-08
	Mevalonic acid*	3.357	2.4749	8.31E-05
	Glyceric acid*	3.8066	2.3755	0.002927
	D-Fructose*	3.8948	2.2424	3.36E-09

	Taurine*	3.4164	2.1733	1.07E-10
	Dimethylglycine*	3.70485	2.0544	2.96E-06
	Dihydrothymine*	3.4872	2.0399	8.89E-08
	Acetylcholine*	3.7295	2.0379	1.91E-11
	Myoinositol	3.52405	1.8804	1.33E-09
	Sucrose	3.8615	1.853	0.001648
	Myoinositol	3.2666	1.7687	4.08E-06
	D-Mannose	3.5613	1.679	4.46E-07
	Mannitol	3.7767	1.6119	0.000132
	D-Serine	3.83825	1.5881	1.55E-06
	Alpha-Hydroxyisobutyric acid	1.3305	1.4265	2.24E-05
	Phenyllactic acid	5.325	1.3211	2.29E-05
	Gluconolactone	4.12005	1.2613	0.00011
	Carbamic acid	8.4607	1.1819	0.004011
	2-Ketobutyric acid	1.0581	-1.0144	0.001956
	Guanidine	2.25325	-1.071	1E-04
	2,2-Dimethylsuccinic acid	2.6763	-1.1025	7.02E-05
	Isobutyric acid	1.0003	-1.1521	8.08E-06
	Methylguanidine	2.8471	-1.2092	7.03E-05
	N-Acetylserotonin	1.45365	-1.3132	8.64E-05
	2-Hydroxycaproic acid	1.7199	-1.3679	3.04E-07
	Oxoglutaric acid	2.97105	-1.8175	3.28E-07
	L-Alpha-aminobutyric acid	1.8899	-1.9721	0.045351
	N-Methyl-a-aminoisobutyric acid*	1.59015	-2.2428	0.022343
	Sarcosine*	2.7409	-2.7234	0.002674
**Co-infection**	Methylguanidine*	3.3654	4.8894	2.68E-12
	Capric acid*	1.33175	3.3899	5.28E-10
	Gluconolactone*	4.121	2.7636	1.75E-11
	D-Mannose*	3.5609	2.581	7.97E-12
	Dihydrothymine*	3.4894	2.3364	0.0276
	Taurine*	3.40125	2.2452	0.000447
	Phenyllactic acid*	5.3251	2.2328	3.90E-10
	1,3-Dimethyluric acid*	3.427	2.2205	0.000654
	L-Glutamine*	3.7471	2.1229	4.66E-06
	D-Fructose*	3.895	2.0717	2.36E-05
	2-Hydroxybutyric acid	4.65975	1.9657	0.024804
	Oxalacetic acid	2.3775	1.9586	0.00051
	Dimethylglycine	3.706	1.8478	0.003769
	Myoinositol	3.2665	1.8339	4.17E-06
	D-Serine	3.83795	1.811	0.00011
	Glyceric acid	3.8058	1.7948	0.000254
	Mannitol	3.7763	1.778	3.90E-07
	Sarcosine	3.5983	1.6489	1.04E-05
	Sucrose	3.86085	1.528	0.003487
	Creatine	3.9209	1.5263	0.051394
	3-Chlorotyrosine	1.48	1.3216	8.35E-05
	N-Methyl-a-aminoisobutyric acid	1.5904	1.2338	0.017176

	Glyceric acid	4.14065	1.2189	0.033353
	Mevalonic acid	3.3273	-1.0046	0.05143
	2,2-Dimethylsuccinic acid	2.67885	-1.0768	5.75E-05
	Beta-Alanine	2.5364	-1.0889	0.088085
	Pyrocatechol	6.89645	-1.1758	0.009328
	Citric acid	2.6449	-1.2131	0.022583
	Sarcosine	2.7126	-1.2884	0.012062
	N-Acetylserotonin*	1.45345	-2.0402	0.034788
	5-Hydroxyindoleacetic acid*	7.1936	-2.1987	0.001535
	Citramalic acid*	2.7561	-2.205	0.003612
	Citric acid*	2.56145	-2.2606	9.67E-08
	L-Alpha-aminobutyric acid*	1.8912	-2.2745	0.017649
	Ethanolamine*	3.1478	-2.3418	7.15E-12
	Gentisic acid*	6.97725	-2.3681	2.88E-06
	Sorbitol*	3.62845	-2.5544	1.98E-08
	Malonic acid*	3.1175	-2.7831	0.01499
	Isobutyryl-L-carnitine*	3.17585	-2.8811	0.008082

Supplementary Table 2. The table represents the results of Volcano plot analyses of infected samples categorized on the basis of age. The significantly regulated compounds with log2(FC) >= ±2 and p-value <= 0.05 upon CHIKV, DENV and Co-infection are represented with asterisk (*) mark.

Samples	Metabolites	ppm	log2(FC)	p-value
**CHIKV (41-70/12-40)**	Valproic acid	1.24975	-1.0806	0.002884
	Trimethylamine*	2.8948	3.4851	0.0242
	L-Glutamic acid	2.33435	1.567	0.051813
	Mevalonic acid	3.351	-2.2998	0.072291
	Citraconic acid	1.9448	-1.0768	0.089595
	Putresine	1.7561	2.4354	0.090933
**DENV (26-51/12-25)**	Capric acid*	1.16805	2.2963	0.006675
	Sarcosine*	2.71045	-4.3463	0.009906
	Adenylsuccinic acid	2.23005	-1.5171	0.022599
	Epi-coprostanol	0.13005	1.2554	0.030404
	4-Hydroxyphenylpyruvic acid	6.88395	1.1577	0.039795
	Tyramine	6.9018	1.0892	0.072705
	Senecioic acid	1.9235	-1.3326	0.073609
**CD (35-60/12-34)**	3-Hydroxyphenylacetic acid*	7.26555	9.7969	0.012505
	4-Pyridoxic acid	7.33635	1.0786	0.013224
	Pyridine*	7.31695	2.2459	0.017587
	Gentisic acid	6.96735	8.6132	0.058617
	Epinephrine	6.93955	4.2365	0.066933

	Atrolactic acid	7.4073	2.5823	0.075974
	4-Hydroxyphenylpyruvic acid	6.88455	2.9307	0.080614
	Quinolinic acid	7.43485	1.911	0.091287
